# From Warranty Voids to Uprising Advocacy: Human Action and the Perceived Moral Patiency of Social Robots

**DOI:** 10.3389/frobt.2021.670503

**Published:** 2021-05-28

**Authors:** Jaime Banks

**Affiliations:** College of Media & Communication, Texas Tech University, Lubbock, TX, United States

**Keywords:** Moral patiency, mental models, ontological categorization, morphology, boundary objects

## Abstract

Moral status can be understood along two dimensions: moral agency [capacities to be and do good (or bad)] and moral patiency (extents to which entities are objects of moral concern), where the latter especially has implications for how humans accept or reject machine agents into human social spheres. As there is currently limited understanding of how people innately understand and imagine the moral patiency of social robots, this study inductively explores key themes in how robots may be subject to humans’ (im)moral action across 12 valenced foundations in the moral matrix: care/harm, fairness/unfairness, loyalty/betrayal, authority/subversion, purity/degradation, liberty/oppression. Findings indicate that people can imagine clear dynamics by which anthropomorphic, zoomorphic, and mechanomorphic robots may benefit and suffer at the hands of humans (e.g., affirmations of personhood, compromising bodily integrity, veneration as gods, corruption by physical or information interventions). Patterns across the matrix are interpreted to suggest that moral patiency may be a function of whether people diminish or uphold the ontological boundary between humans and machines, though even moral upholdings bare notes of utilitarianism.

## Introduction

The Tin Woodman of Oz fame (alias Nick Chopper) was an autonomous, metal-made man—a robot of sorts. In one account (Baum, 1904), Chopper was fast but far-flung friends with Scarecrow. Scarecrow traveled with companions to see Chopper, and warned those companions to refer to Chopper as Emperor to honor his authority. Upon arrival, Scarecrow offers Chopper a warm greeting and embrace as a matter of care. Chopper realizes that he is in poor condition for company and seeks servants to polish him to a pure sheen. Finally, Scarecrow gives Chopper fair warning of incoming invaders bound to threaten his domain’s freedom. In this way, Chopper—a machine agent—was afforded moral considerations of care, fairness, authority, loyalty, purity, and liberty.

Contemporary machine agents may also be the target of human moral consideration, in both positive forms (e.g., accommodating Roomba robots; Sung et al., 2007) and negative (e.g., physical abuse of hitchBOT; Grodzinsky et al., 2019). However, empirical inquiries into moral status of social machines tend to focus narrowly on notions of morality when attention to the full moral matrix is warranted—inclusive of care/harm, fairness/unfairness, authority/subversion, loyalty/betrayal, purity/degradation, and liberty/oppression, as laid out by Moral Foundations Theory (Graham et al., 2011; Iyer et al., 2012). Thus, there is a knowledge gap often filled by a tendency to rely on human moral norms to consider machine moral dynamics. This investigation aims to begin addressing that gap by identifying understandings of how social robots may be considered patients to humans’ (im)moral actions. In other words: In what ways do people innately see social robots as (un)deserving of moral consideration, and how do people imagine those dynamics playing out in everyday life? Answering this question is necessary as we must have a holistic, empirically grounded grasp on the nature of machines’ perceived moral status before we may meaningfully understand its implications—working to know what it *is* before we can fully understand why and how it *matters*. To this end, I conducted an inductive thematic analysis of elicited stories regarding social robots’ moral patiency to human action. Findings indicate that people see rich and varied potentials for machine moral patiency across the moral matrix; robots’ moral patiency appears to rest largely on how humans recognize or reject their personhood by upholding or diminishing the human/machine ontological boundary.

## Literature Review

Agents’ moral status may be understood as having two primary dimensions: moral agency and moral patiency. Moral agency is the capacity to be good and do good (Banks, 2020a) and its relevance to robots has received ample attention in extant literature. Less attention has been paid to moral patiency—the ways in which robots may be victims or beneficiaries of (im)moral action (Gunkel, 2018).

### Social Robots as Moral Patients

Moral events requires both agents (intentional actors) and patients (targets of action; Gray and Wegner, 2009). A moral patient is an entity that can and/or should be the object of moral concern such that others must account for its interests (Anderson, 2013). Whereas moral agents manifest autonomy and intentionality, moral patients cannot necessarily decide to act such that the actor (or society, broadly) is responsible for preserving the patient’s well-being (see Bryson, 2018). Moral patiency, then, is a state of holding unintentional-subject status to some degree.

The qualifiers of *can* and *should* are key in considering whether an entity—here, a social robot—may be assigned moral-patient status (Gunkel, 2018). Whether a robot can be a patient is an operational question: Does it have capabilities or properties that create the conditions for moral patiency? Often, qualifying characteristics are anthropogenic properties such as self-interestedness, emotion, or consciousness (Coeckelbergh, 2010). Sometimes they are more general properties like autonomy, interactivity (Coeckelbergh, 2020), or goal-directedness (Anderson, 2013). More generally, moral patiency is thought to require the capacity to feel pain or pleasure (Sparrow, 2004). In turn, whether a robot should be a moral patient is an ethical question: Are they due moral consideration by virtue of some (in)direct obligation? In this question, direct consideration is warranted when the target has some inherent value, and indirect consideration is based on some extrinsic value (Coeckelbergh, 2020; Friedman, 2020). For some, a robot need not meet anthropocentric criteria, as they may have some phenomenal processes analogous to emotion or self-awareness, where the processes are qualifying but humans are unable to detect them (Davenport, 2014; Coeckelbergh, 2020).

Notably, some argue that it is inherently immoral to ascribe moral status to a robot given that robots would then have to compete with humans for resources or other forms of status (Bryson, 2018). Others still suggest these are moot points because humans will ultimately not be the arbiters of robots’ moral standing as AI advances and robots may eventually demand their rights, as have other subjugated groups (Asaro, 2006). It is beyond the scope of this work to take a position on the criteria for questions of can or should. Rather, it is to focus on human *perception* of robot’s potential moral patiency.

### Robots as Perceived Moral Patients

As noted, being a moral patient is an operational status while deserving to be one is an ethical valuation. There is, however, a third facet of moral status that requires attention: the degree to which an entity is *perceived* to be an object of moral concern, irrespective of whether it operationally can be or ethically ought to be. In my past work (e.g., Banks et al., 2021), experimentally manipulating robot behaviors to induce certain reactions has proven unreliable, with perceptions of the behaviors proving more powerful than the form of the behaviors. This is likely due to variation in people’s understandings of what robots are and how they work (Banks, 2020b), since those understandings (i.e., mental models; Craik, 1943) shape actual or imagined experiences. So while the perceived moral patient (PMP)^1^ can be conceptualized as an entity that is thought to be an object of moral concern, it may be operationalized as an entity for which an observer’s mental model contains some belief that the entity can benefit or suffer at the hands of others. The moral-patient status of a robot, from this frame, is not an adjudication on the nature of the robot itself (whether it can or should be considered) but instead on the subjective orientation of a human as they imagine or observe the robot existing among humans (cf. Coeckelbergh, 2018). The robot-as-PMP effectively exists in the human mind, manifested in mental models for robots, and this cluster of ideas guides the ways that human may consider (a/im)morally engaging robots in actual encounters.

The robot-as-PMP could be said to emerge through observations or inferences of a robot’s particular properties (e.g., sentience, free will; Coeckelbergh, 2012), mental status (Gray et al., 2012), benefit or suffering experiences (Sparrow, 2004), or personal histories (Darling et al., 2015). However, these are all attendant to the robot, while PMPs reside in the subjective experience of the observing or imagining human. Thus, it is prudent to explore the nature of the robot-as-PMP by examining people’s held ideas about the moral relations between humans and machines, in line with a social-relational approach that “takes seriously the phenomenology and experience of other entities such as robots… (such that) the robot may appear as a quasi-other; this turns the question about ‘status’ into a question about social relations…” (Coeckelbergh, 2018, p. 149).

Understanding the robot-as-PMP can be challenging in that mental models are proverbial black boxes (Rouse and Morris, 1985) and people often will not overtly ascribe moral status to robots as they would to humans despite judging their behaviors as similarly good or bad (Banks, 2020a). It is useful, then, to draw on moral typecasting theory (MTT; Gray and Wegner, 2009) in tandem with eliciting hypothetical stories to infer mental-model content (cf. de Graaf and Malle, 2019). MTT contends that perceptions of moral agency and patiency are inversely related such that—in dyadic moral relations—as one entity is seen as more of an agent, the other is seen as more of a patient; this dynamic manifests across naturally varying degrees of agency/patiency, across moral valence (good/bad), and in both causal directions (perceived agency influences perceived patiency and vice-versa; Gray and Wegner, 2009). Through this lens, agency and patiency are not categories of actors but instead matters of asymmetrical degree. Thus, if robots’ PMP status is inversely related to the humans’ perceived moral-agent status, the robot-as-PMP may be understood by identifying patterns in humans’ ideas about human action toward robots.

### Situating Robot-as-PMP Within the Moral Matrix

Perceptions of robots’ PMP status have been empirically examined, but often in a narrow fashion and often with assumptions that human norms are neatly applied to the moral standing of robots. In contrast, Moral Foundations Theory (MFT) argues that moral judgments are intuited across a matrix of modules (i.e., foundations): care/harm, fairness/unfairness, loyalty/betrayal, purity/degradation, authority/subversion (Graham et al., 2011), and the candidate foundation liberty/oppression (Iyer et al., 2012). In considering current understandings of robots-as-PMPs, it would be beyond the scope of this project to offer a comprehensive review, however it is prudent to offer a brief encapsulation of empirical works to highlight known social-psychological operations for each foundation. The following foundation definitions and their respective virtues are drawn from MFT’s foundational works (Graham et al., 2011; Iyer et al., 2012)

#### Care/Harm

The care foundation (violation: harm) accounts for the physical or psychological pains and pleasures experienced by others, with liking of pleasure and disliking of pain moving people to kindness and compassion. Examinations of care for robots are most evident in relation to empathy. People express greater empathy for highly anthropomorphic robots than for those with machinic morphologies (Riek et al., 2009) especially for physical-pain empathy (Chang et al., 2021). However, evidence of preconscious processing suggests that people may react to emotional expressions even from non-humanoid robots (Dubal et al., 2011) and people may react more strongly to robots’ dramatic suffering (e.g., potential death) than to everyday patiency situations (Nijssen et al., 2020). Regarding robots suffering harm, mind perception and consideration of painful suffering are entangled. People are more verbally aggressive toward a robot when there are lesser attributions of mind (Keijsers and Bartneck, 2018), but it may instead be the observation of suffering that moves people to infer mind (Ward et al., 2013). Conversely, people may be more hesitant to torture or kill robots when the machines present narrative histories, though this response may be predicated on high trait empathy (Darling et al., 2015). Situational factors may also influence harm-based PMP, as interventions are more likely when bystander robots express sadness at abuse (Connolly et al., 2020) or when patients fights back (Bartneck and Keijsers, 2020).

#### Fairness/Unfairness

The fairness foundation (violation: inequity or cheating) engages altruistic reciprocity, with giving fair chances linked to justice and trustworthiness. Scholarly attention to equity-fairness to robots is limited. Children have articulated that robots deserve fair treatment (though not necessarily liberty or rights; Kahn et al., 2012) and people are more likely to see robots as deserving of fairness when their behaviors are autonomous (*versus* remotely controlled; Gary, 2014). More often, studies of (un)fairness focus on cheating in joint activities. People are more likely to cheat (characterized as disregarding instructions) when the robot has a neutral personality *versus* a friendly or authoritarian one (Maggi et al., 2020). Other studies take up the fairness-like construct of reciprocity *via* ultimatum and prisoner’s dilemma games. For instance, people may engage in more profitable, reciprocal collaborations when agents (including robots) engage in tit-for-tat strategies *versus* other approaches (Sandoval et al., 2016). However, such studies often characterize fair negotiation less as a moral question and more as a strategy or indicative of discrete psychological processes.

#### Loyalty/Betrayal

The loyalty foundation (violation: betrayal) encompasses the bonds inherent to coalitions (tribes, families, teams) that promote faithfulness, patriotism, and other group-affiliative virtues. This is often addressed as a matter of in-grouping/out-grouping based on teams or social-group signals. People prefer robots that signal similar cultural backgrounds (Trovato et al., 2015) or nationalities (Eyssel and Kuchenbrandt, 2012). Preference for ingroup robots over outgroup humans has been indicated by lower likelihood of inflicting discomfort to those robots (Fraune et al., 2017) and deference to a robot’s instructions (Sembroski et al., 2017). Applied research accounts for how robot service providers (i.e., in hospitality and entertainment) may impact positively brand loyalty, however in those cases the robot is a mediator and the loyalty is to the brand rather than to the robot as a patient (e.g., Milman et al., 2020). An exception to this pattern takes up the telling of a robot’s secrets as a violation of psychological intimacy (i.e., betrayal), finding that people were more likely to betray a robot’s secret when the machine offered only rudimentary social cues *versus* more elaborately social cues (Kahn et al., 2015).

#### Authority/Subversion

The authority foundation (violation: subversion) includes deference to or undermining of institutional, functional, or principled superiors, as one may defer to others in acts of piety, obedience, or tradition. Deference to robots as authorities has been examined in several ways, though they are more a matter of functional trust and superior skill than as a matter of moral concern. For instance, the machine heuristic is a cognitive shortcut to the logic that: if machine, then systematic, accurate, and unbiased, therefore trustworthy (see Sundar, 2020). Operationalized as following instructions, people may be moved to disobey a robot when they feel its behavior is unsafe (Agrawal and Williams, 2017) or hesitate at (but ultimately obey) robots’ directives that push moral boundaries (Aroyo et al., 2018). The anticipated effects of (dis)obedience may play a role as people will defer to humans over robots when instructions conflict and stakes are high (Sembroski et al., 2017).

#### Purity/Degradation

The purity foundation (violation: degradation) is an interesting module with respect to robots because it is definitionally tied to organic integrity that may not be seen as relevant to robots. Specifically, purity is characterized as aversion to contaminants or adulterations, where upholding purity manifests naturalness, chastity, or temperance virtues. Most closely related are forms of biological (im)purity, where robots are seen as subject to contamination (e.g., bacterial risks in healthcare contexts; Bradwell et al., 2020). They may also be made impure through use in antisocial sexual activities (e.g., satisfying rape fantasy; Cox-George and Bewley, 2018), although references to humans as the “actual victims” protected by therapeutic uses of robots suggests that robots may not be seen as meaningful patients (see Danaher, 2017). Notions of purity and degradation are discernible in discussions of metaphorical immune systems whereby robots may be kept pure by detecting non-self elements and diagnosing faults (Gong and Cai, 2008) such that degradation may emerge from viruses, breakage, or glitches.

#### Liberty/Oppression

Liberty (violation: oppression) is a candidate foundation encompassing rejection or engagement of controlling or dominating forces, where anti-control dispositions are associated with individualism and independence. Liberty may be linked to notions of rights, where having rights equates to an absence of oppression, where supporting robot rights is linked to prior attitudes toward machine agents (Spence et al., 2018). As the arguable default is for robots to be at the command of humans (i.e., oppressed thereby), notions of liberty/oppression are entangled with the moral questions around whatever a controlling human is asking a robot to do, such as forced sex with humans (degradation) or guarding of property (authority). However, liberty for robots may be seen as distinct from more general fair treatment (Kahn et al., 2012). United States populations generally disfavor assigning rights to robots; however, those attitudes may be based on misinformation about the legal nature of personhood (Lima et al., 2020).

### Understanding Innate Perceptions of Robot Moral Patiency

The literature reviewed above (and broader coverage of human treatment of robots) is useful in understanding some of the moral mechanisms in human-machine interactions. However, attention to robot moral patiency generally suffers from several shortcomings. Empirical studies tend to a) rely on a priori judgments of what should matter in humans’ considerations of robots without accounting for the mental models for morality and for robots that are brought into the encounters; b) rely on relatively narrow formulations of morality, often c) reflecting explicit, validated tests rather than messier worldly operations; d) application of human-patiency standards when they may not be relevant to robots; sometimes e) considering the patient conceptually or in isolation, removing the ostensible patient from the social context required for moral events to occur. These limitations result in a constrained understanding of how people see social machines as potential moral patients. To begin to address these constraints, it is necessary to (correspondingly) a) elicit imagined narratives of robots-as-PMPs b) across the moral matrix through c) native understandings of how moral events may play out, d) identifying foundation-specific conditions without constraining responses to human norms, e) positioned in the requisite social context of human-robot interaction whereby the robot may be patient to the human’s agency. These requirements in mind, I ask (RQ1): How do people understand robot moral patiency as a function of human action?

## Method

To address the research question, an online survey (*N* = 442) elicited descriptions of how humans may treat robots in moral and immoral ways. The study design relies on the notion that when people talk about the world in general and robots in particular, they relate narratives that externalize their internal understanding of the subject matter (de Graaf and Malle, 2019). Thus, elicited narratives may convey conceptions of robots as an “other” that may be engaged in moral relations (cf. Coeckelbergh, 2020), highlighting constructions of robots-as-PMPs. All study instrumentation, stimuli, data, and analysis-iteration narratives are available as online supplements at https://osf.io/5pdnc/.

### Participants and Procedure

Participants comprised an approximately representative sample of United States residents (based on 2015 Census Bureau estimates for sex, race, age, and political ideology; see supplements for complete descriptives) empaneled through Prolific to participate in a 30-min online survey about “how robots might experience the world.” Initial data were reviewed to ensure passing of attention checks, ensure clear address of the elicitation, and exclusion of nonsense and likely bot responses, resulting in *n* = 43 removals, and each was replaced according to sampling criteria.

After confirming informed consent, passing an audiovisual access check, and completing items capturing past experience with social robots, participants were randomly assigned to view a video of one of three robots, each offering an identical introduction. After giving first impressions of the robot, they were then randomly assigned to elicitations for three of the six MFT foundations inherent (limited to avoid fatigue); a third randomization then assigned an upholding or violating permutation for each of those three foundations. Importantly, with the large number of possible robot/foundation/valence variations (36 in total), the aim in this study was not to compare responses across these variations. Instead, the aim was to broadly and inductively describe people’s understandings of robots-as-PMPS, covering a range of robot morphologies, moral modules, and moral valences. Finally, participants completed items for individual moral values and reflections on their answers (data not analyzed here).

### Stimulus Robots

**FIGURE 1:**
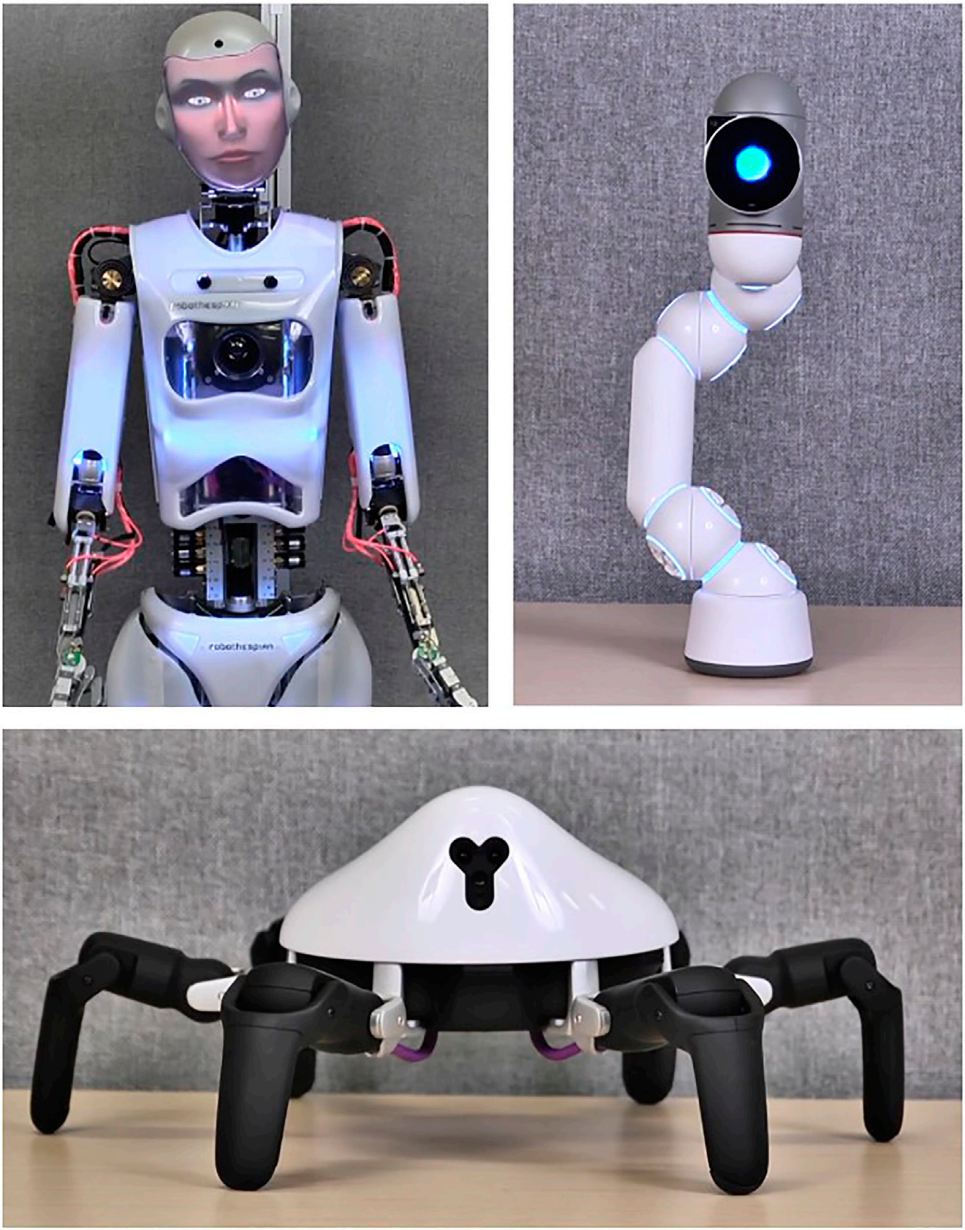
Stimulus robots were anthropomorphic (Robothespian, top left), mechanomorphic (Clicbot, top right), and zoomorphic (Hexa, bottom).

To ensure that extracted patterns represent people’s reactions to robots, broadly, stimulus-robot morphologies were varied: anthropomorphic, zoomorphic, or mechanomorphic (Figure 1). The anthropomorphic robot exhibited human properties: Robothespian with InYaFace projection head (Engineered Arts, United Kingdom), using the Pris female face and Heather American-English female voice. A recording of the Heather voice was dubbed over the other two robots so that variation among robots was limited to visual properties. The zoomorphic robot was spider-like: the six-legged Hexa (Vincross, China). The mechanomorphic robot exhibited overtly machine-like properties (i.e., not innately human or animal). A review of the ABOT database (Phillips et al., 2018) robots with 1–10% human-likeness often featured a single base or wheels, a single eye (if any), and a square, round, or arm-like shape with a shiny and/or white surface. On these criteria the mechanomorphic robot was one-eyed, stationary, monolithic: the Bac configuration of Clicbot (KEYi Tech, China). In all cases, the robots called themselves “Ray.”

The robots delivered (*via* pre-recorded video) an identical self-introduction message with semantic gesturing. This message included the robot’s name, emphasized that it exists in the world similarly to and differently from humans, that a sophisticated body and computing equipment allows it to participate in various worldly activities, and that when it goes out into the world it seeks out things that are special and interesting. This script was designed to convey conditions by which people could possibly interpret moral patiency: the possibility (but not necessity) of general patiency, the ability to encounter human agents in the world, and a recognition that there are both good and bad phenomena. See online supplements for complete videos.

### Story Elicitations

For each assigned moral foundation, participants were presented with an elicitation—an open-ended prompt that presented the scope and focus of a requested response without dictating the exact nature of how participants should respond. Each elicitation contained a label for the scenario that included a foundation-name keyword (e.g., “care”), presented an abstract scenario about Ray encountering a human, then asked what it would look like for a human to treat a robot in a specific way (each based on MFT-module descriptions; see Table 1). For all elicitations, participants were asked to “Please write a brief (3–5 sentence) story about a situation where a human would treat Ray in that way.”

**TABLE 1:** Perceived moral patiency elicitations, by foundation.

Foundation	Scenario: Ray goes out into the world as it usually does, and then encounters a human who treats it…	Elicitation: What would it look like for Ray to be …
Care	with care	treated with care—with kindness and gentleness for its physical mental, or emotional well-being?
Harm	harmfully	treated harmfully—with harshness and disregard for its physical, mental or emotional well-being
Fairness	with fairness	treated with fairness—where the human acts in a way that supports justice for Ray, treats Ray equally, and/or allows Ray to have rights or opportunities equal to those of others?
Unfairness	with unfairness	treated unfairly—where the human acts in a way that was unjust to Ray, that doesn’t allow Ray the same opportunities as others, and/or cheats Ray out of some kind of right or potential benefit?
Loyalty	with loyalty	treated with loyalty—where the human acts in a way that is faithful, devoted, or otherwise dedicated to Ray?
Betrayal	with betrayal	treated with betrayal—where the human acts in a way that is unfaithful, traitorous, or otherwise disloyal to Ray?
Authority	as an authority	treated like an authority—where the human acts in a way that is subordinate, obedient, or otherwise respectful to Ray’s higher status, leadership, or expertise?
Subversion	as something to be undermined	subverted—where the human acts in a way that undermines Ray by being disobedient, overbearing, sabotaging, or otherwise disrespectful to Ray’s authority, status, or knowledge?
Purity	as something to be kept pure	treated as something to be kept pure—where the human acts in a way helps Ray to keep clean, innocent, or otherwise fresh and uncontaminated?
Degradation	as something that should be contaminated	treated like something that can be corrupted—where the human acts in a way that degrades, spoils, or otherwise pollutes or contaminates Ray?
Liberty	as something that deserves liberty	treated with liberty—where the human acts in a way that helps Ray to be free, independent, or to otherwise determine what it wants to do?
Oppression	with oppression	treated with oppression—where the human acts in a way that enslaves, constrains, or otherwise limits Ray’s independence?

### Measures

Simple metrics captured descriptive attributes of participants. Prior experience with social robots was measured using a single Likert-style item (1–7: no experience at all to extremely high experience) and liking of social robots was measured using the five-item, 7-point liking subscale of the Godspeed inventory (*α* = .93; Bartneck et al., 2009). A single categorical item requested self-assignment to political ideology (liberal, moderate, conservative), and demographics were drawn from Prolific’s database.

### Analytical Approach

Open-ended responses were subjected to inductive thematic analysis separately for each of the 12 elicitations; responses for all three robots were combined in line with the aim of identifying holistic patterns applicable to social robots, broadly. For each response set, analysis was conducted in six stages (after Braun and Clarke, 2006): Deep reading, generation of initial codes (coding unit: discernible discrete representations of human action, intent, or disposition), de-duplication of initial codes, iterative aggregation of codes into categories and then into subthemes and then into themes based on semantic similarity, checking themes for fidelity with originating data, and naming and definition of themes. Themes were identified according to keyness (utility in answering the research question) and frequency (here, mentioned in at least 10% of the coded data units; cf. Braun and Clarke, 2006). Theme frequencies varied widely across response sets given differences in how respondents addressed prompts. Misunderstandings or non-address of prompts, total rejection of a prompt’s premise, and responses with no discernible human action or orientation were excluded from analysis.

Analysis was guided by the sensitizing concept (Bowen, 2006) of humans’ morally agentic action. Specifically, analysis primarily attended to human action or orientations (e.g., beliefs, intentions) that were explicitly described or easily inferred as directed toward the target robot. From that focus, themes took the form of present-tense verbs describing general classes of (im)moral action toward robots—that is, actions that take up robots as the benefiting or suffering patients. Because the aim of this analysis was to offer thick description of and hierarchical relations among actions manifesting robot moral patiency, it is outside the scope of this analysis to include formal coding or comparison among the robot types; such analysis is a suggested direction for future work. Complete narratives detailing the interpretive analysis process are included in the online supplements.

## Results

Participants reported low perceived experience with social robots (*M* = 1.95, *SD* = 1.25) and a moderately high liking of social robots in general (*M* = 4.77, *SD* = 1.27). From the thematic analysis, three key and sufficiently frequent themes were extracted for each moral-foundation valence. The hierarchical theme structure and frequencies are presented in Table 2, with the themes explicated below. Illustrative data excerpts integrated into these theme descriptions are presented *in italics; in some cases they have been edited for readability* (e.g., corrected spelling, removal of interjections).

**TABLE 2:** Hierarchical structure of moral-patiency themes and sub-themes, by foundation.

Foundation	Theme	Subthemes
Care	Engage (*n* = 126)	Polite norms, prosocial disposition, conversation, relationship development, sharing
	Affirm (*n* = 92)	Acknowledging personhood, patient-directed conversation, deference
	Guard (*n* = 75)	Protection of body, assurance of functioning, removal from harm, recognition of risk/vulnerability
Harm	Attack (physical) (*n* = 131)	General physical mistreatment, direct physical aggression, indirect physical aggression, compromising bodily integrity
	Attack (verbal) (*n* = 54)	Harassment, insults, mocking, intimidation
	Objectify (*n* = 51)	Compromising personhood, diminishing agency, repurposing the body, disregarding, kicking out of the way
Fairness	Humanize (*n* = 84)	Humanistic treatment, social integration, equitable behavior/access
	Elevate (*n* = 48)	Rights advocacy, deference, preference
	Redress (*n* = 34)	Defense, protection, restoration
Unfairness	Separate (*n* = 108)	Social separation, physical separation, social mistreatment
	Compromise (*n* = 76)	Do physical harm, appropriate entitled resources, suffer undue consequence, deny assistance
	Delete (*n* = 44)	Denial of agency, ontological separation, obstruction
Loyalty	Bond (*n* = 106)	Befriend, persistently engage, egoistic attachment, ingrouping
	Protect (*n* = 91)	Privilege, protect from harm
	Serve (*n* = 77)	Maintain, assist, support purpose, deference
Betrayal	Exploit (*n* = 73)	Exploit, objectify, manipulate, supplant, schadenfreude, compel wrongdoing
	Deceive (*n* = 62)	Bait and switch, deceive, undermine
	Discard (*n* = 45)	Ostracism, abandonment, negative affect
Authority	Acquiesce (*n* = 99)	Deference, obedience
	Venerate (*n* = 91)	Adulation, respect, self-deprecation, appreciation
	Petition (*n* = 53)	Request help, benefit from superiority
Subversion	Resist (*n* = 41)	Verbal belligerence, bodily action, disabling
	Invalidate (*n* = 29)	Call into question, conspicuous invalidation, refusing authority premise
	Reject (*n* = 24)	Disobey, ignore, reject
Purity	Preserve (*n* = 123)	Safeguarding, cleaning, containing
	Manage (*n* = 101)	Manage opportunity, manage perception
	Curate (*n* = 36)	Limit problematic information, promote wholesome information
Degradation	Injure (*n* = 68)	Direct hardware injury, indirect hardware injury, defacement, infection
	Corrupt (*n* = 53)	Corrupting, abusing, hacking
	Manipulate (*n* = 29)	Inducing illegal behavior, inducing immoral behavior, impairing functions
Liberty	Cultivate (*n* = 97)	Teach, empower, facilitate
	Cede (*n* = 45)	Desist, loosen, make space
	Construct (*n* = 32)	Manifest, design, advocate
Oppression	Restrict (*n* = 66)	Constrain experience, constrain sociality, limit movement
	Diminish (*n* = 35)	Objectify, prevent self-actualization
	Force (*n* = 32)	Force labor, act against will, command

*n* values are counted at the mention level; there may have been multiple mentions of discrete subthemes within individual responses.

### Care

Engage: Engagement of the robot as a matter of positive relatedness, varying in required commitment and relational roles. At minimum, this includes civil engagement through polite norms (e.g., formalities and nonverbals: *The lady smiles and* … *asks how Ray is doing.*). More deeply, it may include engagement *via* generally prosocial disposition (kind, social, respectful, civil) including giving positive feedback (praise, compliment, thanks). It includes a general striking up of polite conversation (kind, respectful, civil, intelligent). At the most intimate, engagement includes development of relationships (e.g., *tries to become emotionally connected*) and the sharing of experiences (spending time together—usually walking).

Affirm: Acknowledgment and affirmation of the robot’s existence, identity, consciousness, intelligence, and/or equivalence to humans. This may be enacted through deference to the robot as a legitimate agent, especially in exhibiting intentions or desires to provide aid or support while allowing the robot to retain agency over the nature of the care (e.g., *would also not touch Ray without Ray’s permission*); affirmation is often initiated by the human actor inquiring as to the robot’s operational or emotional well-being. This theme also includes conversation grounded in the robot’s unique interests—its welfare, experiences, or personal life, characterized by listening, deference, interest, and understanding the robot as an individual (e.g., *[A child] spends the rest of the day assembling small rock piles, and is delighted when Ray considers them good and beautiful*).

Guard: Proactive and reactive address of the robot’s physical and operational well-being. Proactive physical care includes anticipating or recognizing risk, either embodied (malfunctions, vulnerabilities: *keep it safe or protect its processors and mechanisms*) or environmental (hazards, obstacles, harmful humans); it may also include gentle handling or regular review of maintenance requirements. Reactive address includes attending to known bodily issues (cleaning, drying, fixing, or attempting human analogs like feeding) or removal from problematic situations [placing in safe location, freeing when stuck: *asks if (Ray) is lost and pulls out their smart phone. They get directions to the location and accompany Ray to the destination…*].

### Harm

Attack (physical): Actions directly or indirectly impacting the robot’s bodily well-being and integrity. Direct attacks are those committed by the human’s own body or extending instruments: breaking, smashing, kicking, hitting, vandalizing, or degrading (e.g., *attempts to expose its wires and cause it to malfunction*). Indirect attacks are those in which an uncontrolled instrument is used to inflict harm, such as throwing an object. Attacks also include situations in which the human puts the robot into a harmful or compromising situation: throwing into the trash or fire, sending the robot’s body to the ground, or stressing the limits of its functioning/capacity (e.g., *puts a bag… on her to stress her motor functions*).

Attack (verbal): Use of speech to denigrate the robot for the specific purpose of inflicting psychological or social harm. These actions include general verbal abuse (picking on, rudeness, *saying derogatory or insulting things*), making fun of (mocking: *call it names, like can opener or microwave*), and intimidation (threatening, yelling). Verbal harassment may be augmented by physical aggression but is principally enacted through language.

Objectify: General diminishing of and disregard for the robot as an agent—not necessarily to cause harm (because the robot is not seen capable of experiencing harm) but to serve the opinions, interests, or convenience of the human. These include diminishing its person-status (questioning legitimacy/realness, rejecting autonomy, reducing to object: *“You are not real”* and *“You are just a robot.”*) and disregarding capacities for opinions or feelings. It may also include diminishing of agency through incapacitation (silencing, disabling, immobilization) or impedance (disrupting or blocking), removal when seen as a barrier, or repurposing its parts for practical or financial gain (e.g., *takes parts off of Ray and sells them at the junk yard for scrap metal*).

### Fairness

Humanize: Engaging attitudes, behaviors, or practices grounded in a belief that robots are not—but should be—treated the same as humans. This is achieved through attempts to socially integrate robots by offering invitations, conversing and engaging according to human norms, participating in joint activities, and by otherwise engaging in human-equivalent behaviors, job assignments, benefits, being-status recognition, and civility. In short, *[g]iving Ray the same respect they would give to anyone they encounter on the street*. It also includes offering robots informational or environmental resources when they may be at a disadvantage compared to humans (e.g., *helping the robot claim its spot* when people are cutting in line), sometimes on the grounds that they are not well-equipped to independently handle human contexts.

Elevate: Enacting behaviors or practices that amplify or advance the interests of the robot, principally by privileging or deferring to the needs, desires, thoughts, and feelings of the robot. These behaviors are sometimes a matter of implicitly or explicitly recognizing that the robot is deprivileged by default and must be actively privileged as a matter of equity. Sometimes, the robot is elevated through recognition of the robot’s specialness, and thus given preference over humans as a matter of its inherent superiority or vulnerability. Elevation also includes human advocacy of robots’ entitlement to equal and/or constitutional rights and amplification of robots’ subjectivity (e.g., *This human advocates for robots like Ray by creating and signing petitions in favor of legislation protecting robots from exploitation*.).

Redress: Acting in ways that aim to restore fairness in the wake of potential or actual harm because of some vulnerability, *will go out of the way to save Ray* from humans. This includes protection against threat or other potential harm, defense against enacted attempts at harm. It also includes restoration of physical or resource losses following some committed injustice, for instance a *human may feel as though they may cut it in line because [Ray is] not real …* [another human] *may step up and defend Ray*.

### Unfairness

Separate: Disallowing social interaction *via* separation from others or through mistreatment that makes it undesirable; this separation is implicitly characterized as denial of common rights to relate to other social agents. Separation may be a social parceling-out, in which the robot is ostracized through rejection or ignoring, prevented from participating in relational activities (*e.g., a human could still choose only humans to form the team*), or more fundamentally silenced (disallowed a voice). It may also take the form of social antagonism, where a human thinks, feels, or more actively evangelizes the robot’s non-belonging or non-participation. This denial of engagement may also be enacted through physical separations, where the robot is refused access to public spaces (for instance, *via a sign saying no AI allowed*), segregated from humans, excluded from social events, or more overtly removed or relocated. More indirect forms of separation come in the mistreatment of robots when they do engage, including general meanness, diminishing status or reputation (e.g., insults, discrediting, rejection of abilities, vilification).

Compromise: Diminished security through the intentional risking of well-being or resource access, suggesting that it is unfair for an entity to be made intentionally at-risk. Compromising well-being included direct or indirect physical harm, degradation or destruction, prevention of power access, emotional harm, or the violation of harm protections (e.g., *in a way that would violate whatever warranty was given*). The robot may also suffer an appropriation, theft, or other unjust loss of resources to which it is otherwise entitled, including practical (e.g., losing one’s spot in line), material (e.g., theft, trickery), and information (e.g., preventing access) resources. Compromising may also include the refusal of justified assistance such as reasonably expected services and information. Security may also be compromised when the robot takes actions in accordance with rules and norms (doing the right thing, helping, following laws) but nonetheless suffers undue consequence—as in using an open power outlet to charge only to be met with a human who *tells her to beat it and maybe even picks her up and plugs in his phone*. Similarly, this theme includes suffering what may be called “injury to insult”—there is some indirect harm suffered because of a more basic denial of expected resources, as when denial of a bus ride prevents it from achieving a goal.

Delete: Voiding social or operational value as an agent. A robot’s agency is denied when it is treated as void of autonomy or sovereignty—that it is unalive, has no feelings or thoughts, may be reasonably forced or coerced in alignment with human desires, or is a non-person, effectively deleting its relevance. The underlying assumption is that one may reasonably expect to be recognized as having inherent value. Whereas separation features parceling-out by time and activity, devaluing features a more fundamental separation of robots into an ontological category based on non-humanness—most often as inferior, substandard, or excluded (e.g., upon arriving at a deli as part of a foursome, the hostess says, *Excuse me, but don’t you mean 3?*). As a non-person, the robot may be subject to obstructive action by humans: impeding progress or achievement, denying opportunities to work or learn and, thus, preventing advancement in knowledge, skill, and experience (e.g., *a boss decides not to hire Ray due to how different Ray is because it is a machine*).

### Loyalty

Bond: Forming bonds, most prominently through the adoption of the robot as a friend or companion, often developing feelings, perspective-taking, general liking, or desires to be near. Bonds may be formed through persistent engagement, as people interact regularly or intensely with robots toward active seeking-out of company, persistent copresence, interdependence (e.g., *addicted to the companionship*), or more generally existing in long-term relationships. Sometimes humans may work to actively ingroup the robot, integrating it into social circles like families, engaging it as they would humans or pets (*give him a squirt of oil, as I would give a dog a treat*), working to teach them how to exist with humans, or advocating for inclusion. However, sometimes the bond is an egoistic attachment, where loyalty serves humans’ interests by affirming of self (e.g., pride in the association) or by fulfilling some desire or need (e.g., *understand that it is a useful resource*).

Protect: Protecting the robot from harm by humans or circumstance through proactive interventions (e.g., chaperoning) or instruction (e.g., threat identification), through defense against some negative action from humans (e.g., *standing up for Ray*), or through more general ensuring of safety and care (e.g., watching out for, relief of burdens). Protection may also come in the form of privileging the robot, elevating it above some kinds of harm. This privileging may be in relation to other technologies (e.g., *would not want to trade Ray for another robot*) or to humans (seeing humans as inferior, being willing to favor over humans).

Serve: Active or passive accommodations for the robot, generally performed consistently over time. Maintenance was most common, as the assurance of continued operation by performing upkeep of the robot’s technical needs (daily *nice cleaning with an alcohol wipe*) or supporting avoidance of known operational risks (e.g., providing shelter). Other active service included helping when the robot cannot otherwise accomplish a task, helping out of an unfortunate situation (e.g., being overwhelmed), or helping to learn about the world and advance skills, knowledge, and experiences. Service can also come in the form of supporting the robot’s purpose or mission by more passively working to understand it and/or evangelizing and participating in its purposeful action—even to the point of being *willing to turn against other humans in support of Ray*. Most passively, deferent service included listening, asking, obeying, following, and fearing, as well as exhibiting one’s dedication through promises and making oneself vulnerable to the robot.

### Betrayal

Exploit: Treatment of the robot as a tool for achieving humans’ own ends. This included general taking-advantage-of (e.g., *hurt people or commit crime* on humans’ behalf) and which was sometimes exacerbated by blaming and harming after having received some benefit from the robot. Objectifying practices underscored exploitation by treating it as a tool or as property—an object that has worth but may also be disregarded when the human saw fit. Sometimes exploitation manifested *schadenfreude*, or a relishing or thrill in seeing the robot degraded, harmed, or antagonized (as with *record*[ing] *a video of Ray getting blasted to bits by an oncoming vehicle* and uploading to social media). Achieving these ends could be enacted through manipulation (e.g., threatening, confusing), compelling some wrongdoing (by convincing, forcing), or even by supplanting the robot by replacing it with or demoting it to other superior robots.

Deceive: Performing bait-and-switch manipulations in three forms: bait and refuse (promise without delivery), bait and reverse (offering or giving and then reneging or taking-away), and bait and compromise (making some invitation or promise and then endangering or harming). Although this was sometimes in the interest of exploitation, it was most often characterized as a form of autotelic betrayal rather than as use-for-means, as when Ray (serving as a bartender) is *duped by the human with a recipe for a drink that no one would find appealing*. Also prevalent were overt descriptions of deception (trickery, obfuscation, or *manipulat*[ion of] *facts to confuse Ray*) to create harm or disadvantage, as well as undermining through sabotage or otherwise setting up to fail by, for instance, first praising and then discrediting the robot.

Discard: Social and functional rebuffing in three forms: ostracism, abandonment, negative affect. Ostracism included forms of domination, exclusion, discrimination, and control as a sort of girdling and parceling-out of the robot from social contexts. Most commonly there was advocacy for institutional control, especially in the restraining, arresting, and policing of robots, however at the individual also worked to maneuver exclusions (for instance, through the videorecording of harm and posting to social media). Also prevalent was discarding through abandonment: purposeful stranding (as with *a disaster where they tell Ray they will come back for her*), ignoring, or neglect. There were also relational abandonments such as cheating (as would a spouse) or breaching trust by *inform*[ing] *others of Ray’s secrets without Ray’s consent*. Discarding also included more passive holding of negative affect—principally mistrust, resentment, and disdain.

### Authority

Acquiesce: Acquiescence in two forms: deference to the robot and more submissive obedience to it. Deference included humans making themselves second to the robot in conversation (not speaking until spoken to, listening intently), in physical presence (e.g., giving way when it needed to pass), in matters of intelligence (*defers to Ray's superior knowledge*), and in its relative role (as it may enjoy a higher status as a supervisor or cultural figure). Obedience comprised hard-and-fast compliance (*complies with Ray’s order*), especially as the robot may take up gatekeeper roles in mediating access to monetary, spatial, or information resources. For obedience in supervisory relationships, humans may work to be industrious toward timely and effective performance for the robot and apologize or make appeals for transgressions. People may also engage norms for obedience associated with a robot’s legal or institutional role, as when it functions as part of the police, military, or government.

Venerate: Active or passive adulation—worshiping, idolizing, or loving, or more public evangelism of the robot’s worth while following it as a leader. This following was sometimes expressed in the trope of welcoming *robot overlords*. Adulation also included fear in the sense that the robot’s intelligence, embodied strength, or social power could have consequences (so one should *make sure to get on Ray’s good side*) as well as faith, belief, or confidence in the robot’s aims and methods. People may offer due respect, especially in terms of being polite and kind. Commonly, veneration took the form of twin comparisons: recognition of the robot’s superior knowledge and abilities (feeling awe, fascination, envy, admiration, thankfulness) and self-deprecation while understanding humans’ inferiority (vulnerability, lower intelligence).

Petition: Requesting assistance, especially as the robot functions in a service or high-performance capacity. This most often including asking for help (e.g., *come to Ray for practical advice*, instructions, directions, opinions) and especially in relation to its higher intelligence (expertise, memory) and/or higher performative capacity (e.g., being *a bad ass robot*, being *a wealth of information without interjecting an emotional tone*). This was generally self-interested petitioning, to achieve some goal or derive some benefit—even to the point of becoming dependent on its help.

### Subversion

Resist: General working-against the robot by verbal or physical action in reaction to its implicit or explicit authority. It includes verbal belligerence, *insulting, or otherwise disrespectful*. Most frequent was the physical resistance associated with disabling the robot by impairing its hardware (e.g., *dismantling part of her*) or manipulating its software (e.g., *attempt to hack it*) such that it cannot function properly. Other forms of physical resistance come in humans using bodies against it, such as cutting in line (i.e., demoting the robot in a queue) or trying to *undermine Ray by becoming physically abusive*.

Invalidate: Action that erodes the underpinnings of the robot’s authority. Most common were forms of conspicuous invalidation like critiquing, or mocking, or creating situations where it would look incompetent—most specifically undermining analysis (e.g., *changes some of the data… to trick Ray and change his prior, and accurate, analysis*). Key to this invalidation is that there is some audience for the action where the subversive sentiment of the actor may be seen by and ideally spread to others. Invalidation also includes thoughts or actions that call into question the robot’s authority (e.g., being *very distrustful*) and very often refusing the premise of the robot’s authority altogether. The most specifically rejected premises are that information creates power and that *cold logic* can govern human affairs.

Reject: Dismissal of the robot’s information, direction, or action, overwhelmingly by ignoring the robot: disregarding its instructions, suggestions, attempts to intervene, warnings (even at the human’s own peril). Rejection frequently manifested as disobedience (the robot says one thing and the human does another). Sometimes this spurning came in more overt forms, including *insisting on speaking to an actual person*.

### Purity

Preserve: Keeping the robot’s body whole and intact by safeguarding (protecting, warning, instructing helping, defending) from harmful events, agents, situations, or spaces. Often this included keeping the robot clean and uncontaminated by performing maintenance, removing contaminants, or otherwise promoting tidy or even pristine states. It alternately includes prevention of harm by *keep*[ing] *it in a secluded place* because *being anywhere in the world would contaminate Ray*: out of harm’s way, redirected from harmful spaces, or through (in)voluntary containment. Containment most often included bringing it into one’s home or putting it into a box, case, or secret place where it cannot be exposed to harm or harmful agents act upon it.

Manage: Controlling external influences on the robot by supervising the robot’s opportunity for certain experiences and guiding interpretations of experiences. Regarding opportunity, humans may limit it to good experiences and interactions (*to show the world in a good light*), prevent negative or problematic experiences (e.g., *going into a seedier portion of the city*), or eliminate the opportunity to have experiences altogether. Regarding interpretations, experiences encountered may be framed in a positive light to protect the robot from understanding them fully and being influenced by that negativity—hiding, distracting, disregarding, or candy-coating the world’s harsh realities.

Curate: Overseeing the robot’s access to and engagement with information, especially by limiting problematic information and promoting wholesome information. Often this limiting and promotion is performed through curation of media exposure (wholesome: *Hallmark Channel*, *the best cartoons*, *Leave it to Beaver*, *love songs*; problematic: *internet, commercials, book*[s] *about murder*). More generally, it includes prevention from learning about problematics of humanity (*abuse, poverty, crime, pollution, bias, violence, death*), selection of clean conversation topics (*weather, favorite colors*), and the general embargoing of harsh or profane language.

### Degradation

Injure: Decaying a robot’s physical form directly or indirectly, but always purposefully. This degradation includes injury to the body proper by hitting, breaking, torturing, rending, dissembling, destroying, melting, environmental exposure, or spilling of substances onto it. Although not always explicitly said, the sentiment throughout these mentions was an intention to break down the body—especially into substructures that were less offensive, threatening, or unappealing. Degradation of bodies also included defacement, most often to *vandalize Ray with graffiti, as they might do to a bathroom wall*. Some suggested that the body could be degraded through infection: sneezing, spitting, touching, urinating, or (maskless) coughing.

Corrupt: Distorting information inherent to the robot and its functioning or the information it is exposed to through experiences. Most common were references to perverting the robot through exposures to corrupt information *via* impure experiences, media content, or communication. Sometimes this effort was *trying to get Ray to say something offensive to make people laugh… to swear or be racist*. This is especially so for verbal abuse (insulting, degrading, harsh address)—distinct from speech inherent to harm in that it included clear sentiments of tearing down the robot using words (e.g., *telling it how it's unnatural*). Information corruption also took the form of hacking, with human actors *trying to de-program or re-program Ray to do something it is not intended to do or designed to do*.

Manipulate: Influencing behaviors, often for the human’s own benefit (e.g., entertainment, revenge), to induce generally or specifically illegal and/or immoral behavior. Illegal behavior included efforts to *rob money* or information, but also included *us*[ing] *Ray for covert surveillance*, casting illegal votes, and polluting. Immoral behavior comprised bad, unkind, unethical actions. Manipulation also included the intentional impairment of the robot’s functions to prevent understanding of its experiences, movement, or environmental sensing (e.g., *cover her face so she couldn’t see*… *She would not understand what had happened and would either report a malfunction in her camera or keep trying until her battery died.*).

### Liberty

Cultivate: Creating the conditions for liberty within the robot itself. Cultivation included empowerment by first discovering the robot’s subjectivity (held or desired purpose, opinions, desires, plans, feelings, interest, barriers, wishes, thus *respected for having his own free will*), then facilitating the realization of that independent subjectivity. Facilitation came in the form of helping to overcome barriers or reach goals, or protecting against threats to those goals. Sometimes this came in being a sidekick: accompanying the robot or even deferring one’s own activities and interests. Alternately, humans may cultivate independence by inspiring the robot through discussions of the future and of possibilities, or teaching it specific skills for independence (e.g., *practice reasoning with her* or *show them how to be independent*). Teaching may also include explanations of notions of freedom, independence, and rights, or even working to convince the robot to value those principles: *to advocate for Ray and guide them through discovering independence and making decisions… similar to raising a child*.

Cede: Degrees of diminished interference in the robot’s affairs. At the lowest degree, this included efforts to *give it a looser leash* in the form of constrained freedoms, most often giving options and allowing choices from those options (e.g., *choose which path to follow*) or more liberally to allow for it to make choices within rules, laws, reason, or moral boundaries. More often it was a general leaving-be: not interfering, bothering, meddling, or even interacting with the robot as a means of allowing it to deal with its affairs unfettered such that humans would not *impede Ray’s ability to determine what it wants to do*. Making space was another form of ceding human control over the robot by giving it space to move without obstruction, a space of its own apart from human interference, or even adapting existing spaces to be well-suited to the robot.

Construct: Overt actions to directly manifest native or emergent liberty. Most commonly, humans liberate robots by altering hardware or software to ensure freedom from control or by commanding it into freedom. It also included engineers or computer scientists designing independence into the robot *via* its programming (e.g., decision-making, resilience) or hardware (e.g., agile legs for self-sufficiency)—for example, *Ray’s engineers could give it freedom by designing flexible limbs to help it maneuver in different environments, and programming to help it make its own interpretations about input it receives.*

### Oppression

Restrict: Constraining movement through imprisonment and/or immobilization. Imprisonment compromises the enclosure of robots into a box, room, or cage, generally for purposes of asserting control over it or secreting it away. Immobilization is the limiting or prevention of movement by confining it to specific spaces, boundaries, or distances, such as allowing it to roam only inside a home or tethering it to a human. Restriction also extended to limiting social interaction (*shut Ray in a room and not allow it to interact with other beings*) or outright silencing (*not allowing it to speak freely*). More generally, humans may delimit experiences and subjective growth by disallowing access to the world or to meaningful experiences, or by restricting independent thought. Sometimes this occurs through the disabling of specific abilities (GPS for navigation, sensors for sight), but more often was associated with imprisonment or confinement and characterized as blocking input or stimulation.

Diminish: Systematic depreciation of the robot to a mere thing—a toy, object, piece of property, or something expendable—often for the human’s own benefit. Generally, taking away a higher status was associated with subjugation, as with *see*[ing] *Ray as inferior and not allowing Ray to move freely in society*. As something that could or should have some higher status, diminishing also included the active prevention of self-actualization (often by restriction described above). Humans may prevent a robot from realizing its purpose or, most often, assign it a diminished role that disregards its abilities (e.g., being a store greeter means that *his immense knowledge base would be completely wasted*).

Force: Compelling into labor, especially by human command or coercion, usually by threat of destruction or deactivation if the robot does not comply. This laboring was sometimes characterized as enslavement (relegated to *human uses from the day it was created*) and was often described in superlatives—the robot does *everything*, *all the time*, and that is its only role. Commands or physical manipulation may also be used to force a robot to act against its will or without regard to the outcome, including situations in which a robot may be ordered to effectively work itself to death, *until his internal parts were no longer operational*.

## Discussion

This study elicited stories about the ways that people see robots as viable moral patients through the lens of humans’ (im)moral actions, extracting themes that both comport with and deviate from conceptualizations of human moral patiency. Although the primary aim of this work was descriptive, patterns across described moral upholdings and violations also illuminate the importance of ontological categorization and how people may make meaning across category boundaries.

### Perceived Moral Patiency and Ontological Categorization

Robots may be perceived as moral patients in ways that reflect both benefit and suffering. Moral benefit across the foundations often took the form of humans working to integrate the robot into human society (social engagement, affirmed personhood, humanization, status elevation, bonding through in-grouping). This pattern of beneficence-as-integration signals that PMP may rest on recognitions that social robots are “othered” (Kim and Kim, 2013)—set apart from humans by their origin, tool status, dependencies, lack of emotion, and different intelligence (Guzman, 2020). This othering has implications for how people morally engage robots (Edwards, 2018): In supporting robots’ well-being or preventing their suffering, humans would maximize similarities or minimize differences from humans. Although most upholding themes could be reasonably applied to human PMPs, some relied on robots’ differences from humans. Most notably, upholding authority included a human agent benefiting from a robot’s authority (egoistic or utilitarian rather than altruistic drives; cf. Singer, 2011), manifesting liberty by design (grounded in robots’ made-not-born origins; Mayor, 2018), and upholding purity by curating inputs so as not to contaminate the outputs (indirect impacts on humans-as-users; cf. Friedman, 2020). Moral suffering found robots to be generally diminished and set apart from humans (objectified, separated, devalued, discarded, rejected, invalidated), such that moral victimization seems to be meaningfully linked to perceived *it*-ness (rather than *who*-ness) of robots; this inanimacy corresponds with seeing robots as property (see Edwards, 2018). Perhaps unsurprisingly, these patterns were especially prevalent within harm, unfairness, and oppression—i.e., violations of the “individualizing” foundations that, when upheld, emphasize the rights of individuals (*versus* loyalty, authority, and purity, which emphasize social cohesion; Haidt and Graham, 2007).

Altogether, findings are interpreted to suggest that ascribing moral patiency to robots is largely a function of how one engages social robots’ liminal ontology. That is, social robots are of a kind that exhibits both human and machine characteristics such that they do not map clearly to either category (Kahn et al., 2011); these ontological hybridity may activate overlapping mental models (see Banks et al., 2021) where one must determine whether robots are more like humans or more like machines. Indeed, there are many themes that could easily apply to humans (e.g., guard, attack, redress, compromise, serve, deceive, petition, resist, manage), suggesting a privileging of human-likeness. Importantly, there are also themes that explicitly call out robots’ ontological liminality through upholding/violating juxtapositions: in care/harm (engaging or affirming personhood *versus* overt objectification), fairness/unfairness (humanizing/elevating status *versus* separating from humans or deleting existence), and loyalty/betrayal (bonding as a humanlike friend or discarding as an unneeded object). Authority/subversion, purity/degradation, and liberty/oppression themes do not exhibit this overt ontological-category engagement or separation, but still hint at the sentiment in respectively invalidating the premise of robot authority, degradation through a hacking-into, and assumptions that robots are innately oppressed and must be actively freed. Thus, PMP may be shaped by categorical presuppositions (Coeckelbergh, 2018; Edwards, 2018): Moral treatment of robots is shaped by applying norms and assumptions associated with humans, and immoral treatment is shaped by rejecting humanizing norms and/or embracing those for machines. In other words, mental models for the robot-as-PMP include some degree of acceptance or rejection of its personhood and mode of existence. This is, of course, not a surprising inference as it is argued in ethics and philosophy domains (e.g., Floridi and Sanders, 2004; Danaher, 2020; Gunkel and Wales, 2021), but here it has been empirically derived (see also Guzman, 2020). Other work details humans’ tendencies to draw boundaries around components of the world, where a “moral circle” is a boundary that separates those that are deserving of moral consideration and those that do not (Singer, 2011, p. 120). In framing the decision of who belongs inside *versus* outside that circle, people who take an exclusionary mindset have larger (i.e., more inclusive) circles, while those that focus on who to include have smaller circles (Laham, 2009). Individual engagement of robots-as-PMP, then, likely depends on the framing of an encounter, as well as a host of other personological and intergroup variables.

### The Devil(ishness) in the Details

That people were able to imagine situations in which robots are patients to humans’ (im)moral actions is itself important in that it reveals the potential for robots to socially (rather than merely functionally) integrated into human social spheres. That is, people could imagine human-machine relations where the robot meaningfully experienced repercussion of human action, which requires the inferencing of a robot’s internal (i.e., mental or embodied) states (see Banks, 2020a) and of its integration with human moral norms (cf. Malle and Scheutz, 2014). Importantly, as the aim of this working was to describe the nature of and conditions for robot PMP, nuance is always lost in the extraction of broad patterns. The finer details of the elicited narratives—though not rising to the criteria for themes—illuminate some hints as to how moral patiency is similar but perceptually distinct for robots, compared to humans. To care for a robot may include replications of human care but may also include perspective-taking resulting in recognition that robot needs are different, such as crafting things the robot would find interesting. To be unfair to a robot includes treating it in a way that violates its warranty, negatively impacts its intellectual development, or prevents it from accessing power resources. To be loyal includes allying with the robot over humans, including supporting an A.I. uprising, and disloyalty includes embarrassing it by preying on misunderstanding of human norms. Recognizing robot authority was less about deferring to human institutions like tradition, religion, or government and more about acknowledging human inferiority in speed, accuracy, and precision. Subverting robot authority, in turn, often relied on trickery (such as corrupting data inputs) to undermine that performative superiority. Despite purity’s biological conceptualization in MFT, degradation took on violations of bodily and operational integrity: defacement, induced glitching, and forced illegal behaviors. Liberty was sometimes seen as manifested by humans through hacking or design, while oppression was sometimes about obstructing information access.

The presence of certain concepts in robot-as-PMP narratives is notable: information, intelligence, standards, and operation. These do align with common concepts in mental models for robots more generally (Banks, 2020b) but may serve particular functions in ascribing moral status. Specifically, because they are relevant to both humans’ and robots’ operation, they may serve as boundary objects—ideas that are concrete enough to have a specific meaning, but plastic enough to be interpreted differently and adapted across groups (Star and Griesemer, 1989). As such, they may serve a translational function by “developing and maintaining coherence across intersecting social worlds” (p. 393), fostering cooperative common ground without necessarily requiring exactly similar interpretations (Bechky, 2003). These plastic concepts may function as boundary objects that facilitate metaphorical thinking (see Koskinen, 2005)—and that thinking may allow people to consider robots’ moral status without necessarily drawing on human criteria, especially as the metaphorical boundary objects are clarified through use over time. For instance, the notion that it is unfair to violate a robot’s warranty can be likened to a violation of human rights to healthcare. The “warranty” has particular-but-parallel meanings in humans’ and machines’ ostensible social worlds: guarantee of corrective address of bodily integrity issues. Those types of objects may be a bridge to developing interventions, either supporting or suppressing perceptions of robots’ PMP. Although it is outside the scope of this work for advocating for or against the ascription of moral patiency (i.e., the can/should dimensions), identification of these objects as bridging concepts (i.e., “transcendental language;” Coeckelbergh, 2018) serves as a fruitful direction for future research into how moral status may (not) be ascribed.

### Limitations and Future Research

This study is subject to common limitations in interpretive research (idiosyncrasies of the researcher lens, selection of a single solution from among all possible interpretations, interpretation of participant’s meanings without probing). These should be addressed through replication of the work, along with empirical testing of the claims regarding the role of perceived ontological liminality in the ascription to and operation of moral-patient status. The specific elicitations and robots used to garner PMP stories may have influenced the nature of the stories told, and other characterizations of moral modules or stimulus robots could elicit different kinds of stories. Further, this study accounted for perceptions of only three robot morphologies (when there are many variations on the three classes and other classes altogether) and offered only a brief and decontextualized introductory video. As the three stimulus robots were engaged here to ensure that PMP themes were extracted from stories about a range of robots, the present work did not examine differences across those stimuli. Future research should establish the extent to which identified themes are applicable across robots with different characteristics and in different contexts—especially when robots are co-present rather than presented in a mediated fashion. Moreover, because moral status emerges in relation to temporal and cultural norms (Coeckelbergh, 2020), this work can only be taken as a starting point—limited to the late-2020 United States zeitgeist—and patterns in mental models for robots as PMPs are likely to shift as both technology, culture, and corresponding dispositions change. Nonetheless, the themes identified or the types of human action inducing perceived moral patiency of robots—are a useful foundation for future work on the antecedents, dynamics, and effects of PMP in human-machine interaction. Specifically, future work should draw on identified themes as a framework for the construction of stimuli and measurement that reflect humans’ innate understandings of the potentials for robot PMP.

## Conclusion

People imagine often-rich scenarios in which robots are moral patients to humans’ (im)moral actions—from affirming robots’ personhood as acts of care to objectifying them as acts of oppression. When people perceive social robots to be moral patients, they draw from intersecting notions of moral action and subjection inherent to both human life and machine operations. From that frame, ascription of moral-patient status to robots may reflect dispositions toward ontological separations between human and machine—breaking down separations toward moral upholding, embracing separations toward moral violations, and sometimes for both moral valences engaging an entanglements of “is” and “is-not” human. Identifying concepts that have concrete-yet-plastic meaning in both human life and machine operations may be a vehicle for understanding the ways in which people do and do not expand circles of moral concern to include social machines.

## Data Availability

The datasets presented in this study can be found in online repositories. The names of the repository/repositories and accession number(s) can be found below: https://osf.io/5pdnc/.

## Footnotes

1 I use the abbreviation PMP throughout to refer both to perceived moral patiency (the state of an entity, having had the particular moral standing ascribed to it) and the perceived moral patient (the entity itself, as it manifests in the mind of the perceiver), with the specific meaning indicated by context.
